# Whole-Genome Sequencing and Phenotypic Analysis of *Streptococcus equi* subsp. *zooepidemicus* Sequence Type 147 Isolated from China

**DOI:** 10.3390/microorganisms12040824

**Published:** 2024-04-19

**Authors:** Yan Su, Zehua Zhang, Li Wang, Baojiang Zhang, Lingling Su

**Affiliations:** 1 Department of Microbiology and Immunology, College of Veterinary Medicine, Xinjiang Agricultural University, Urumqi 830052, China; 2 Xinjiang Academy of Animal Science, Urumqi 830000, China

**Keywords:** *Streptococcus equi* subsp. *zooepidemicus*, whole-genome sequencing, prophage, hyaluronidase gene

## Abstract

*Streptococcus equi* subsp. *zooepidemicus* (*S. zooepidemicus*) is one of the important zoonotic and opportunistic pathogens. In recent years, there has been growing evidence that supports the potential role of *S. zooepidemicus* in severe diseases in horses and other animals, including humans. Furthermore, the clinical isolation and drug resistance rates of *S. zooepidemicus* have been increasing yearly, leading to interest in its in-depth genomic analysis. In order to deepen the understanding of the *S. zooepidemicus* characteristics and genomic features, we investigated the genomic islands, mobile genetic elements, virulence and resistance genes, and phenotype of *S. zooepidemicus* strain ZHZ 211 (ST147), isolated from an equine farm in China. We obtained a 2.18 Mb, high-quality chromosome and found eight genomic islands. According to a comparative genomic investigation with other reference strains, ZHZ 211 has more virulence factors, like an iron uptake system, adherence, exoenzymes, and antiphagocytosis. More interestingly, ZHZ 211 has acquired a mobile genetic element (MGE), prophage Ph01, which was found to be in the chromosome of this strain and included two hyaluronidase (*hyl*) genes, important virulence factors of the strain. Moreover, two transposons and two virulence (*virD4*) genes were found to be located in the same genome island of ZHZ 211. In vitro phenotypic results showed that ZHZ 211 grows faster and is resistant to clarithromycin, enrofloxacin, and sulfonamides. The higher biofilm-forming capabilities of ZHZ 211 may provide a competitive advantage for survival in its niche. The results expand our understanding of the genomic, pathogenicity, and resistance characterization of *Streptococcus zooepidemicus* and facilitate further exploration of its molecular pathogenic mechanism.

## 1. Introduction

*Streptococcus equi* subsp. *zooepidemicus* (*S. zooepidemicus* (SEZ)), a member of Lancefield group C *streptococcus* and an important opportunistic pathogen that primarily affects the equine and swine industries, causes a wide spectrum of infections, like pneumonia, pleuropneumonia, endometritis, infertility, and strangles-like diseases [1,2,3]. SEZ has no obvious host restrictions and may infect cows, dogs, sheep, birds, cats, monkeys, and humans [4,5]. Virulent strains of *S. zooepidemicus* were linked to disease outbreaks that killed over 300,000 pigs in China in 1970. The first disease outbreak in swine due to *S. zooepidemicus* in Germany was reported in 2023 [6]. In the past few years, several cases of SEZ hemorrhagic pneumonia characterized by a high mortality have been reported in dogs [7]. Moreover, there have been many cases of SEZ meningitis, septicemia, and arthritis in humans who have frequently been in close contact with infected animals or animal products [8,9,10,11]. SEZ-induced purulent pericarditis was also reported in a human who was infected following contact with horses [12]. As such, SEZ is a pathogen of concern in many countries around the world.

*Streptococcus equi* subsp. *equi* (*S. equi*) strain 4047 (Se 4047) (ST179) was identified from a strangled horse in New Forest, England, in 1990 [13]. In the year 2000, SEZ strain H70 (Sz H70) (ST147) was identified in a healthy racehorse in Newmarket. ATCC35246 (Sz 35246) (ST194) was isolated from a deceased pig in China’s Sichuan province [14]. Strain MGCS10565 (Sz 10565) was recovered from a human case in Brazil in 1998 [15], while strain BHS5 (Sz BHS5) was obtained from a dog with acute fatal hemorrhagic pneumonia in London, the United Kingdom, in 2001 [16]. Based on these strains, researchers discovered that *S. equi* and *S. zooepidemicus* are both pyogenic *streptococci*, like the significant human pathogen *Streptococcus pyogenes*. These bacteria share several virulence pathways and demonstrate cross-species horizontal DNA exchange [13,17].

Previous research has helped us understand some of the genetic and pathogenic properties of *S. equi* strain 4047 (Se4047) and *S. zooepidemicus* strain H70 (SzH70). Holden et al. analyzed the entire genomes of Se4047 and SzH70 and discovered that *S. equi* pathogenic specialization was generated by a combination of gene loss and gene gain through the acquisition of mobile genetic elements (MGEs). Furthermore, other studies indicated that the acquisition of four prophage-encoded superantigen genes played a significant role in the development of Se4047 [16,18].

Recently, third-generation sequencing has been increasingly used to perform pathogen comparative genomics [19], which has elucidated many novel virulence factors and pathogen–host interaction mechanisms [20,21]. Whole-genome sequencing (WGS) and the subsequent comparative genomic analysis have become very powerful tools that have allowed investigations of the movement of virulence and other host adaptation genes between *streptococci* isolates from different horse farms [22,23]. Moreover, mobile genomic elements (MGEs) were found to promote the spread of virulence and antibiotic genes. The mediation of MGEs often leads to bacterial strain diversification and also alters phenotypes. MGEs make up around 15% of every *S. aureus* genome and play an important role in host adaptation and pathogenicity [24,25]. Moreover, the Pathogenwatch cgMLST web bioresource is available for tailored genomic analyses of populations of S. equi and its close relative, *S. equi* subspecies zooepidemicus, recovered from horses and other animals, including humans, worldwide.

The genetic relationships among all 670 *S. equi* isolates were recently determined, revealing the national and international transmission events driving this endemic disease in horse populations worldwide, published by Mitchell et al. [26]. However, the mechanisms that contribute to the genetic diversity of SEZ and how the virulence factor influences the severity of illness in infected animals are still poorly understood. Furthermore, the particular mechanisms of transmission have yet to be thoroughly and systematically investigated utilizing genome-wide profiling.

*S. zooepidemicus* could pose a threat to humans, and studying its genomic features is imperative to understanding its antibiotic resistance and virulence. This study sequenced the *S. zooepidemicus* strain ZHZ 211 genome to determine the virulence and antibiotic resistance genes, as well as to define its genetic population structure. Contrary to individual genotypic characterization reports, we aimed to obtain an overall insight into the genomic architecture of *S. zooepidemicus* using whole-genome sequencing and phenotypic analysis.

## 2. Materials and Methods

### 2.1. Bacterial Strains and Growth Conditions

In this investigation, *S. zooepidemicus* s ZHZ 211 and control strain *S. equi* XJ 5012 were isolated from the same farm in Xinjiang, China. Both strains were cultured at 37 °C in Todd Hewitt broth (THB).

Overnight cultures starting from a single colony were diluted in 500 L THB to an OD600 of 0.02. After incubating the plates at 37 °C for 24 h, the OD600 of each sample was determined at 2, 4, 6, 8, 10, 12, 14, 16, 18, 20, and 24 h. At least three separate experiments were carried out, each with three technical replicates.

### 2.2. DNA Preparation and Genome Sequencing, Assembly, and Annotations

*S. zooepidemicus* strain ZHZ 211 and *S. equi* strain XJ 5012 were grown overnight in THB at 37 °C to achieve the mid-logarithmic phase for the WGS. Following the manufacturer’s instructions, chromosome DNA from two strains (ZHZ 211 and XJ 5012) was extracted from a single colony of pure bacterial culture using the TIANam Bacteria DNA kit (Tiangen, Beijing, China). NanoDrop (Thermo Scientific, Waltham, MA, USA) was used to measure DNA. Electrophoresis using 0.8% agarose gels was used to determine DNA quality and RNA contamination.

Whole-genome sequence data for two strains were generated by a combination of PacBio Sequel II (Pacific Biosciences, Menlo Park, CA, USA) and the Illumina HiSeq platform (Illumina, San Diego, CA, USA). For Illumina sequencing, at least 1 μg genomic DNA was used for each strain in the sequencing-library construction. DNA samples were sheared into 400–500 bp fragments using a Covaris M220 Focused Acoustic Shearer following the manufacturer’s protocol. Illumina sequencing libraries were prepared from the sheared fragments using the NEXTflex™ Rapid DNA-Seq Kit. 

For PacBio sequencing, DNA fragments were purified, end-repaired, and ligated with SMRT bell-sequencing adapters following the manufacturer’s recommendations (Pacific Biosciences, Menlo Park, CA, USA). The resulting sequencing library was purified three times using 0.45× volumes of Agencourt AMPure XP beads (Beckman Coulter Genomics, Danvers, MA, USA), following the manufacturer’s recommendations. Next, a ~10 kb insert library was prepared, and the 10 kb library for each strain was sequenced using the PacBio RSII platform with two SMRT cells for each isolate using standard methods.

Fastp v0.23.0 was used to quality-filter the raw Illumina sequencing reads produced from the paired-end library. SMRT Analysis v2.3.0 was used to process the raw sequencing reads produced by the PacBio platform. Prokka v1.13.3 [27] was used for genome annotation. ISEs-can v1.7.2 [28] was used to predict insertion sequences. PHASTER [29] and Islandviewer4 [30] were used to forecast prophage areas and genomic islands, respectively. The CRISPR Recognition Tool v1.1 [31] was used to predict CRISPR. IslandViewer [32] discovered genomic islands (GIs). CGView [33] was used to create the genome atlas. Antibiotic resistance genes were annotated using the Comprehensive Antibiotic Resistance Database (CARD). Virulence factors were predicted by using BLAST to search against the Virulence Factor Database (VFDB).

### 2.3. Comparative Genome and COG Analyses

The previously published complete genome sequences of *S. equi* strain Se 4047 and *S. zooepidemicus* strain H70 that were obtained from the National Center for Biotechnology Information were used as the reference strains for comparative genomic analyses. Multiple genome alignments of the four strains used Blast Ring Image Generator (BRIG) software [34] and progressive Mauve [35]. In order to compare the numbers of shared and unique orthologous genes, VennDiagram [36] was used to generate the Venn plots of *S. equi* XJ 5012 (ST179), *S. zooepidemicus* ZHZ 211 (ST147), *S. equi* strain Se 4047 (ST179), and *S. zooepidemicus* strain H70 (ST147). We further analyzed the Cluster of Orthologous Group (COG) categories of the proteins using eggNOG and compared them with the referenced strains *S. zooepidemicus* H70, ATCC35246, NCTC12091, and NCTC6176 and *S. equi* 4047.

### 2.4. Antimicrobial Susceptibility and Biofilm Formation Assay

To determine whether the antimicrobial resistance genes in the ZHZ 211 genomes conferred the predicted resistance, antimicrobial susceptibility testing was carried out using the disk diffusion method according to the Clinical and Laboratory Standards Institute guidelines [37], and susceptibility testing was performed for 21 antibiotics altogether: amoxicillin; ampicillin; cefuroxime; ceftiofur; cefoxitin; penicillin; gentamicin; streptomycin; erythromycin; clarithromycin; doxycycline; oxytetracycline; tetracycline; levofloxacin; norfloxacin; enrofloxacin; ciprofloxacin; sulfafurazole; sulfadiazine sodium; rifampin; and clindamycin. The control strain for the susceptibility experiments was the *Escherichia coli* strain ATCC 25922.

Briefly, 20 μL of ZHZ 211 and XJ 5012 bacterial log-phase cultures were added to the wells of 96-well flat-bottom microliter plates (Greiner, Nürtingen, Germany) containing 200 μL of THB medium with 1% fibrinogen. Plates were incubated at 37 °C for 24 h and 48 h without agitation. The medium alone served as a negative control. The medium was removed, and then washed three times with 200 μL of PBS (pH 7.0). The remaining biofilm was fixed with 200 μL of methanol for 10 min and was then stained with 1% (*w*/*v*) crystal violet in 100 μL for 10 min. The wells were washed with PBS five times and dried for two hours. After adding 200 μL of 95% ethanol (*v*/*v*) to each well, the plate was shaken for 10 min to release the stain from the biofilms, and a 595 nm value was measured with a Tecan GENios Plus microplate reader (Tecan, Grödig, Austria) [38]. All assays were performed in triplicate and repeated three times, starting with new cultures.

### 2.5. Infection Experiments for S. zooepidemicus Strain ZHZ 211

BALB/c mice aged 6–8 weeks were purchased from the Xinjiang Medical University experiment animal facility in Urumqi, Xinjiang, China. Animal studies were carried out in accordance with the Animal Ethics Committee of Xinjiang Agricultural University in China. The protocols for this animal research were authorized by the Xinjiang Agricultural University Committee on the Ethics of Animal Experiments (Urumqi, Xinjiang, China; Protocol Permit Number: 2108002). All animal tests were designed to cause as little pain to the mice as possible.

BALB/c mice were subcutaneously injected with 5 × 10^8^, 1 × 10^9^, and 2.5 × 10^9^ CFU of *S. equi* strain XJ 5012 and *S. zooepidemicus* strain ZHZ 211. The mice were monitored for 10 days to determine their survival statuses, and the variations in survival were plotted using Kaplan–Meier curves and assessed using the log-rank test. The lungs and spleens of infected mice were homogenized and diluted suitably for bacterial load analysis, and bacteria colonies were counted on TH agar.

### 2.6. Statistical Analysis

We repeated each assay three times independently and applied a one-way ANOVA test to assess the differences between groups using GraphPad Prism software 9 (GraphPad, La Jolla, CA, USA). Virulence comparisons were analyzed using a two-tailed, non-parametric Student’s *t*-test or a one-way ANOVA test. Data are presented as means ± SEMs or as geometric means. Log-rank (Mantel–Cox) tests were used to compare the survival between groups of mice. A value of *p* < 0.05 was considered statistically significant.

## 3. Results

### 3.1. Genome Sequencing and Genomic Features of ZHZ 211 Strain

**Figure 1:**
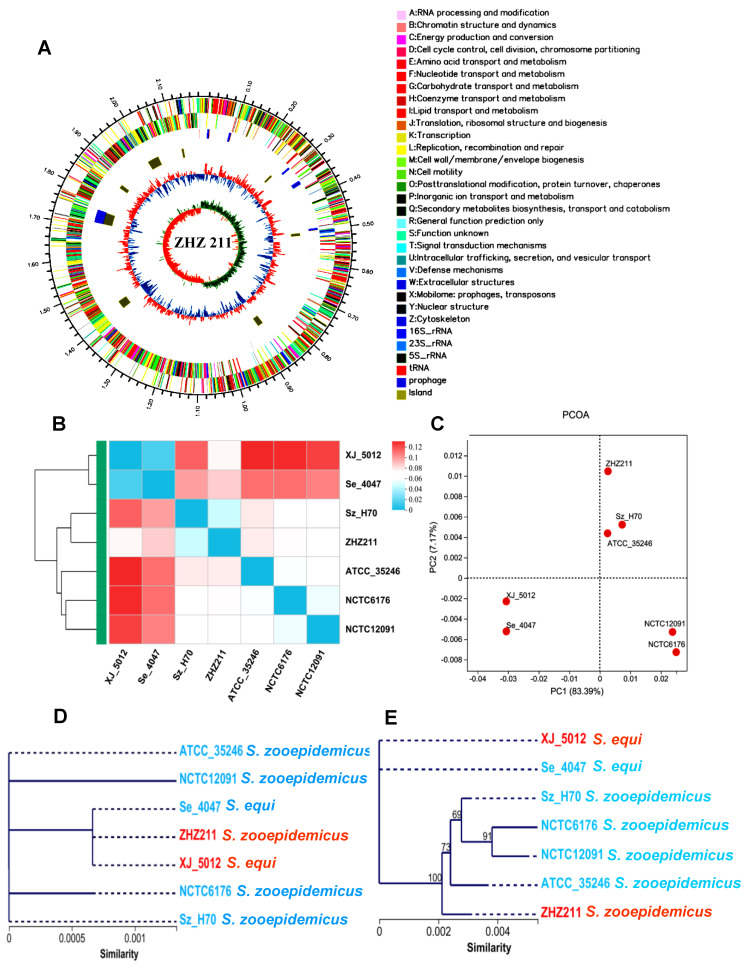
Circos diagram, heatmap of COG function, and phylogeny of ZHZ 211 and other 7 reference strains. (**A**) Circos map of ZHZ 211. (**B**) Heatmap based on the COG function of ZHZ 211 and other six *streptococci* reference strain genomes. (**C**) PCOA (principal component analysis) of 211 ZHZ genomes and six reference strain genomes based on COG function. (**D**) Phylogenetic analysis of the *16S rRNA* gene sequences of ZHZ 211 isolated in this study and seven related reference strains. (**E**) The phylogenomic tree was constructed based on housekeeping genes of seven related reference strains.

We acquired and assembled PacBio data for *S. zooepidemicus* strain ZHZ 211. The size of the ZHZ 211 isolate genome was 2,185,322 bp, and the G + C contents were 41.3%. The predicted number of open reading frames was 2205, with an average length of 848 bp. Our data reveal that ZHZ 211 has one prophage (Ph 01 (41,917 bp)) and XJ 5012 has three prophages (Ph 01, Ph 02, and Ph 03 (53,998 bp, 40,815 bp, 101,029 bp)). We observed a genome island (GI) in ZHZ 211 (8 GI) compared to the XJ 5012 (13 GI) strain (Figure 1A).

We further analyzed the Cluster of Orthologous Group (COG) categories of the proteins using eggNOG and compared them with the referenced strains *S. zooepidemicus* H70, ATCC35246, NCTC12091, and NCTC6176 and *S. equi* 4047. The in silico functional COG annotation of the genes in these seven strains revealed their possible different functions and metabolic fitness between the phylogroups. The COG heatmap of these stains indicated that the COG function of ZHZ 211 was more related to *S. equi* XJ 5012 (Figure 1B). The principal component analysis (PCOA) showed that ZHZ 211 was clustered with H70 and ATCC 35246 in one group, and that XJ 5012 and strain 4047 were clustered in another group (Figure 1C). Phylogenetic analysis based on *16S rRNA* revealed that ZHZ 211 and XJ 5012 are closely related strains (Figure 1D), and the phylogenetic results based on the housekeeping genes of these strains indicated that ZHZ 211 and H70 are closely related (Figure 1E), which is consistent with the results of the COG function analysis.

### 3.2. Genomic Synteny and Core Genome Analyses

**Figure 2:**
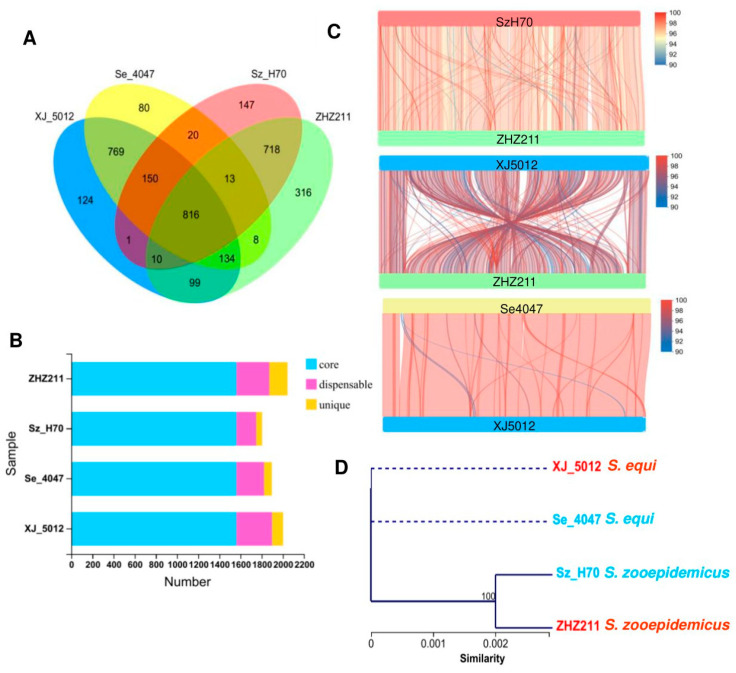
Venn diagram and synteny analysis of the genomes of ZHZ 211 and 3 reference strains. (**A**) Venn diagram showing the number of shared and unique orthologous genes among the four strains of ZHZ 211 and three reference strains, XJ 5012, Se 4047, and Sz H70. (**B**) Histogram of the number of core, dispensable, and unique genes among four ZHZ 211 strains and three reference strains, XJ 5012, Se 4047, and Sz H70. Assigned in Cluster of Orthologous Group (COG) categories. (**C**) Collinear genome alignment of ZHZ 211 and 3 reference strains. (**D**) The phylogenomic tree was constructed based on housekeeping genes of 4 strains.

The pangenome is classified into three categories: core genes (found in all strains), unique genes (found in just one isolate), and dispensable genes (found in more than one isolate). As shown in Figure 2A,B, the four pangenomes of the strains ZHZ 211, XJ 5012, Sz H70, and Se 4047 consisted of 1562 core genes. Furthermore, the pangenome of the ZHZ 211 strain (173) has more distinct genes than the other three isolates (XJ 5012 106, Sz H70 57, and Se 4047 75), whereas XJ 5012 (336) has more adaptable genes than the other three isolates (ZHZ 211 311, Sz H70 185, and Se 4047 260). 

To explore the genetic differences between the ZHZ 211, XJ 5012, Sz H70, and Se 4047 strains, their genomes were analyzed for genomic synteny. As shown in Figure 2C, there are a higher number of genome inversions and rearrangements between the strains ZHZ 211 and XJ 5012, whereas the XJ 5012, Se 4047, ZHZ 211, and Sz H70 strains show a high degree of synteny, which is consistent with the phylogenetic results based on the housekeeping genes of these four strains (Figure 2D).

### 3.3. Comparative Genome Analysis and Prophages of ZHZ 211

**Figure 3:**
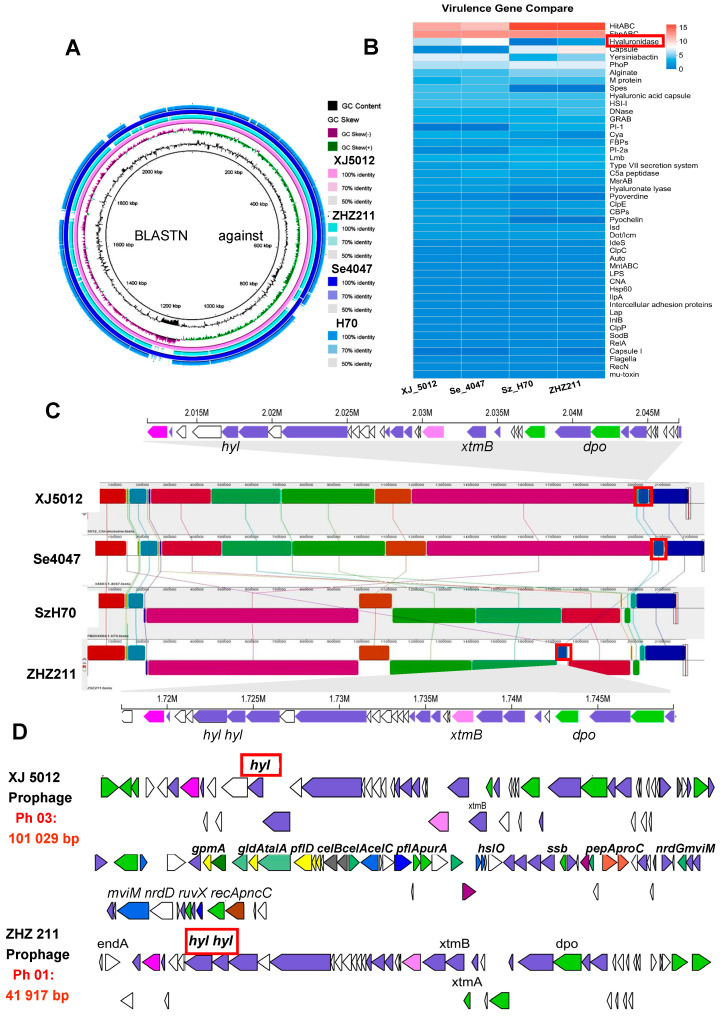
Comparative genomic analysis of *S. zooepidemicus* ZHZ 211 with three related reference strains. (**A**) The circular comparison images of XJ 5012 (central reference) with three strains (ZHZ 211, H70, Se 4047) regarding the large chromosome. (**B**) Heatmap of the distributions and numbers of virulence genes detected among the ZHZ 211 and 3 reference strains. Squares with different colors represent numbers or presence of virulence genes. (**C**) Genome alignment of ZHZ 211 and 3 reference strains (XJ 5012, H70, Se 4047) using progressive Mauve software (1.2.0). Boxes with the same color indicate the syntenic regions. (**D**) The gene cluster of prophages of ZHZ 211 (Ph 01) and XJ 5012 (Ph 03).

The genome of the ZHZ 211 was compared and aligned to the genomes of the *S. equi* XJ 5012 isolates *S. zooepidemicus* H70 (Sz70) and *S. equi* 4047 (Se 4047) to examine their collinear regions (Figure 3A). Then, the comparison results of the numbers and distributions of virulence genes of the four strains (ZHZ 211, XJ 5012, Sz H70, and Se 4047) revealed that ZHZ has more defensive virulence genes, like *ClpC* (1), *Alginate* (5), Grab (3), *MsrAB* (2), *ClpE* (2), *MntAB* (1), *SodB* (1), *ClpP* (1), and *RelN* (1).

The genome synteny was explored by comparing the linear organization of the two isolates with the other reference strains, *S. zooepidemicus* H70 and *S*. *equi* 4047. One block of genes in strain ZHZ 211 was related closely with the other *S. equi* strains Se 4047 and XJ 5012 (Figure 3B), and the prophages were located on this block. This result indicated that the gene might be horizontally transferred by prophages. Different numbers of virulence (hyaluronidase) genes were observed among the four strains (Sz70, ZHZ 211, Se 4047, and XJ 5012) (Figure 3C).

Prophages are significant in their virulence. The ZHZ 211 strain and reference strain XJ 5012 genomes were examined for the presence of prophages. The strain ZHZ 211 carried one intact prophage of a 41.9 kb (Ph 01) size with 39.68% GC contents, and strain XJ 5012 carried three intact prophages of 101 kb (Ph 01), 54 kb (Ph 02), and 40.8 kb (Ph 03) sizes with 40.23% 40.04%, and 39.79% GC contents. In further analysis, two virulence-related genes (hyaluronidase (*hyl*)) were found in the prophage (Ph 01) of ZHZ 211, with the sizes of 1905 bp and 1101 bp. One virulence gene (*hyl*) was found in the prophage of XJ 5012 (1119 bp) (Figure 3D). 

**Figure 4:**
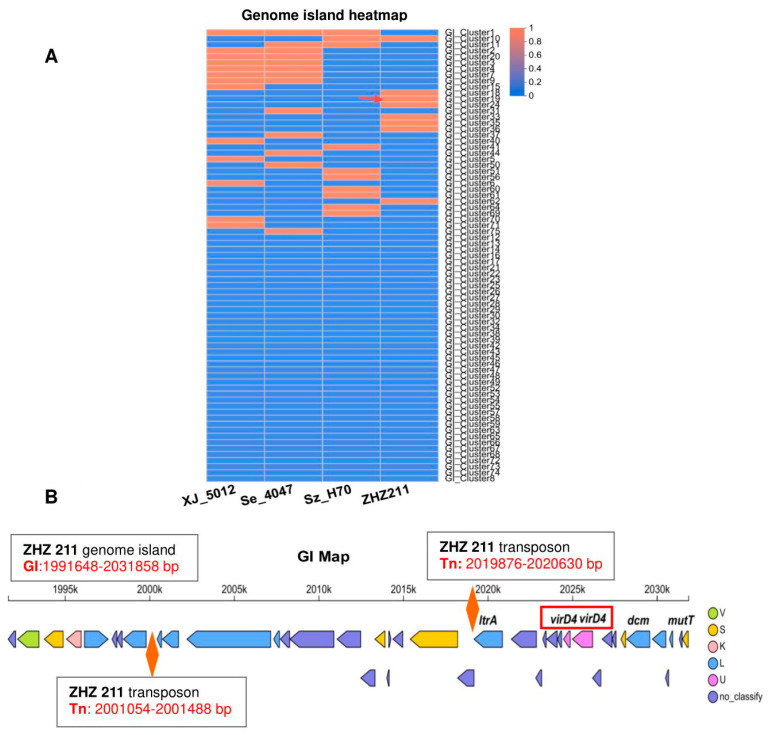
Comparative analysis of genome islands of ZHZ 211. (**A**) Heatmap of numbers and distributions of genomic islands in genomes of ZHZ 211, H70, Se 4047, and XJ 5012. Different colors of squares represent the numbers or existence of genomic islands. (**B**) The schematic diagram of gene clusters of genome island (GI: 1,991,648–2,031,858 bp) and two transposons of strain ZHZ 211.

Over the entire chromosome of ZHZ 211, there are eight genome islands (GIs) encoding 184 genes (Figure 4A). As GIs play an important role in the virulence of SEZ, we focused on the elements of the GIs in further detail. Interestingly, we revealed that one GI (GI: 1,991,648–2,031,858 bp) of the HT321 contains two transposon (Tn) regions and two copies of *virD4* gene cassettes (Figure 4B).

### 3.4. Growth, Antimicrobial Resistance Gene, Phenotype, and Biofilm Formation of ZHZ 211

**Figure 5:**
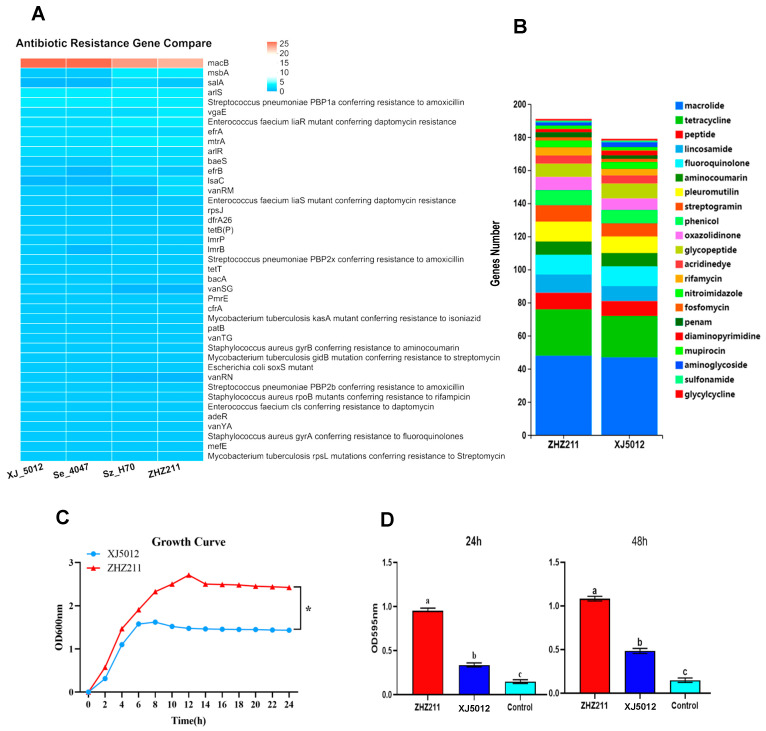
Analysis of antibiotic resistance genes, growth, and biofilm quantification assays for ZHZ 211 and control strain XJ 5012. (**A**) Antibiotic resistance gene comparison of ZHZ 211 and three reference strains. (**B**) Comparison of numbers of antibiotic resistance genes of two strains. (**C**) ZHZ 211 and XJ 5012 were cultured in THB broth at 37 ℃. The OD600 of the culture was measured every two hours. The data represent the means and standard deviations of the results of three independent experiments (* = *p* < 0.05). (**D**) Biofilm formation was evaluated by monitoring the A at 595 nm after crystal violet staining of bacterial cultures after 24 and 48 h. The data shown represent the means and SDs of three independent experiments. There is a significant difference (*p* < 0.05) between numbers with different letters; no significant difference (*p* > 0.05) exists between numbers with the same letters.

The two tested strains carried multiple antimicrobial resistance genes, but the numbers and distributions of these genes were different. The antibiotic resistance gene number of ZHZ 211 is 192, which is higher than the 179 of XJ 5012. ZHZ 211 has the fewest macrolide antibiotic genes (32) among these four strains (Figure 5A,B), including *macB* (23), *mtrA* (3), *efrA* (2), *imrp* (1), *mefE* (1), *efrA* (1), and *cfrA* (1).

Among the 21 antibiotics, the *S. zooepidemicus* strain ZHZ 211 exhibited resistance to seven antibiotics, including ampicillin, cefuroxime, penicillin, clarithromycin, enrofloxacin, sulfafurazole, and sulfadiazine. *S. equi* strain XJ 5012 displayed resistance to six antibiotics, including cefuroxime, gentamicin, clarithromycin, enrofloxacin, doxycycline, and tetracycline. Both strains were susceptible to cefuroxime, clarithromycin, and enrofloxacin (Table 1). There is no doubt that antimicrobial resistance acquired through genetic mechanisms is the major reason for the resistance of many pathogens.

In the growth-rate-dynamic experiments, the ZHZ 211 strain showed a higher growth rate than the XJ 5012 isolate (Figure 5C). We aimed to understand whether there is a relationship between the resistance phenotypes of the ZHZ 211 and XJ 5012 strains and their ability to form biofilm. While significant differences in biofilm formation were observed between the two isolates, the biofilm-forming ability of ZHZ 211 was significantly higher than that of XJ 5012 (*p* < 0.01) (Figure 5D), indicating that there is a correlation between the biofilm formation by the two isolates and their antimicrobial resistance phenotypes.

### 3.5. Virulence Genes and Virulence-Associated Phenotypes

**Figure 6:**
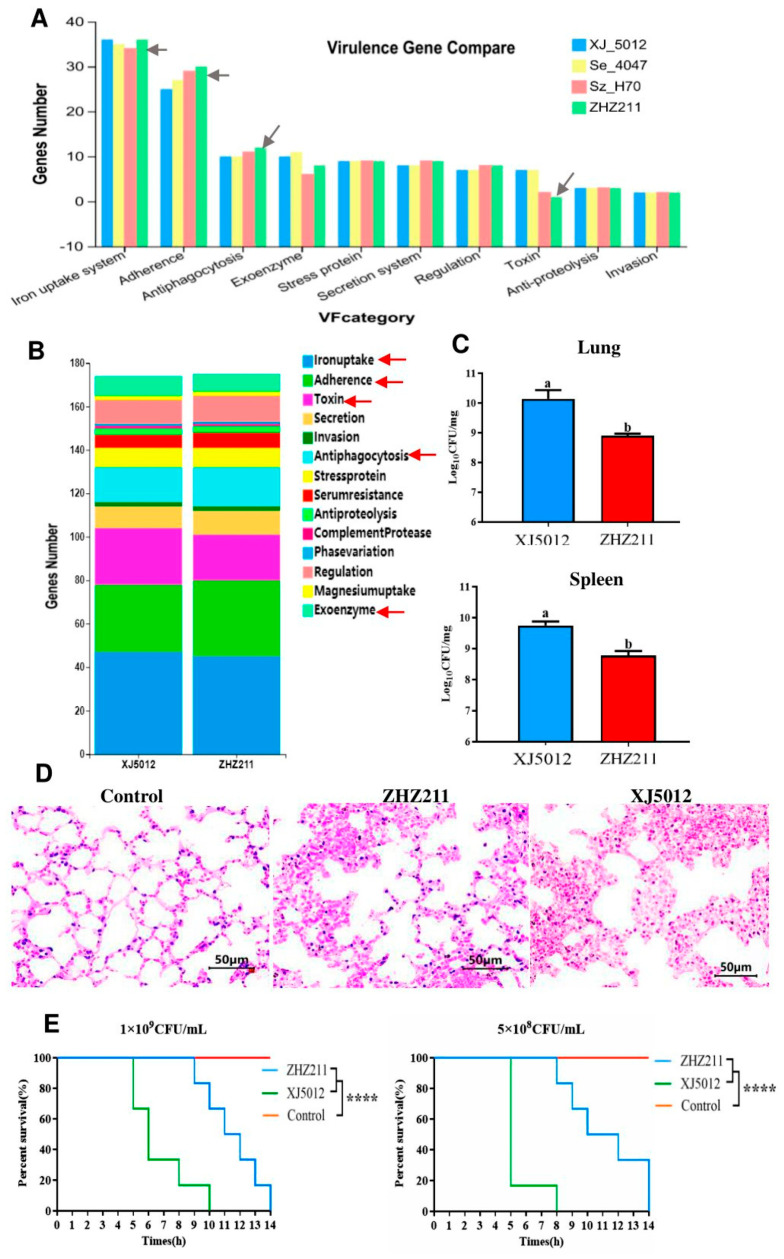
The virulence analysis of ZHZ 211 and reference strain XJ 5012. (**A**) Comparison of the distributions and numbers of virulence genes in ZHZ 211 and 3 reference strains. (**B**) Distribution of nonspecific virulence genes in two isolate genomes. (**C**) Virulences of two isolates in a mouse infection model. Mice were injected intraperitoneally with 5 × 10^8^ and 1 × 10^9^ CFU of XJ 5012 or ZHZ 211. At 14 h post-infection, the lung samples in PBS were plated onto THB, and colonies were expressed as log10 CFU/mg. The data represent the means and standard deviations of the results of three independent experiments. Statistical analysis was performed using the unpaired *t*-test. There is a significant difference (*p* < 0.05) between numbers with different letters; no significant difference (*p* > 0.05) exists between numbers with the same letters. (**D**) The lung tissues of mice were subjected to HE staining. (**E**) Survival rates of mice post-challenge (**** = *p* ≤ 0.0001).

To evaluate the virulence characteristics and the correlation between the virulence factors and virulence-associated phenotypes of ZHZ 211 and XJ 5012, we further tested the virulences of the two isolates. Ten categories of virulence genes were identified in the four strains (ZHZ 211, XJ 5012, Sz H70, and Se 4047) and the virulence gene numbers of the four strains were compared. It was found that the ZHZ 211 strain has more iron uptake systems, adherence, and antiphagocytosis genes and the fewest toxin genes (Figure 6A). A total of 174 virulence factor-encoding genes were identified in the two isolates (Figure 6B). Although the numbers of virulence genes are the same, it was found that the ZHZ 211 strain has more adherence (35 vs. 31), antiphagocytosis (18 vs. 16), and serum resistance (7 vs. 6) genes. However, XJ 5012 possesses more iron uptake systems (47 vs. 45), toxin genes (26 vs. 21), and exoenzyme genes (9 vs. 8).

In a mouse infection model, mice inoculated with strains ZHZ 211 and XJ 5012 (5 × 10^8^ CFU and 1 × 10^9^ CFU) died over a period of 14 days. The XJ 5012 isolates showed higher lethality than the ZHZ 211 strain. Mice spleens and lungs infected with ZHZ 211 had lower bacterial loads than those infected with XJ 5012 (Figure 6C), indicating that ZHZ 211 had lower virulence. Mice lung tissues of ZHZ 211- and XJ 5012-infected mice were fixed for histopathology analysis, and the HE assays revealed that both lung tissues were damaged upon the challenge and exhibited thickened alveolar septa (Figure 6D). Moreover, ZHZ 211 caused low lethality and damage in the mice (Figure 6E). 

## 4. Discussion

*S. zooepidemicus* is usually considered a commensal organism in the pharynx, oral cavity, and respiratory system of horses, leading to severe respiratory diseases [24,25], as well as abscesses, neonatal septicemia, and endometritis [39]. Recently, severe clinical disease in horses and a wide range of animal species has been significantly associated with *S. zooepidemicus.* Moreover, the population of the *S. zooepidemicus* strain is highly diversified and performing the genome sequencing and evaluation of its properties are crucial for meeting the needs for characterizing the bacterial strains and pathogenicity. Recently, in 2019, the first outbreaks of sudden deaths, increased mortality, and increased abortion rates associated with *S. zooepidemicus* in pigs housed in commercial facilities in North America were reported. The whole-genome characterization was published by Costa [40]. In this study, *S. zooepidemicus* ZHZ 211 isolated from an equine farm was comprehensively investigated by comparative genomic analysis and in vitro characterization.

The formation of biofilms allows microbial pathogens to create a safe sanctuary in which bacteria remain in a protected environment. Many bacteria and pathogens utilize a biofilm strategy to survive inhospitable conditions and cause disease, including *Listeria* monocytogenes, *Salmonella, Shigella, Staphylococcus aureus*, and *Escherichia coli* [41,42,43,44,45]. Wang et al. reported that biofilm formation in *S. suis* causes a reduction in virulence due to the downregulation of virulence factors, such as CPS [46]. Our study is consistent with this finding; the ZHZ 211 strain with a higher biofilm-formation capability showed lower pathogenicity in a mouse model compared to the reference strain XJ 5012, which had a lower ability to form biofilms.

Previous research has shown that bacteria in biofilm have increased resistance to antimicrobial agents [47]. Many recent studies have also suggested a link between the biofilm formation ability and bacterial virulence [45,46,47]. Takeshi [48] reported that biofilm-forming *prevotella intermedia* strain 17 exhibited a stronger ability to induce abscesses in mice than the biofilm-negative strain 17-2. In the present study, the virulent ZHZ 211 strains had a greater ability to form biofilms and were more resistant to antibiotics than the *S. equi* strain XJ 5012 based on their susceptibility profiles against 21 antimicrobials. 

The influence of high bacterial numbers in biofilms on antimicrobial resistance in vivo and in vitro has been reported [49,50,51,52]. According to our result, the importance of non-inherited resistance should be noted, mainly concerning infections that are associated with biofilm production, especially for some infections wherein a high number of *streptococci* cells can accumulate at the site of infection [53]. Here, we showed that the ZHZ 211 strain grew faster than the control strain, displayed a stronger biofilm production ability, and was resistant to more antimicrobials than XJ 5012.

*Streptococci* contain various types of mobile genetic elements, including transposons (Tns), insertion sequences (ISs), integrative conjugative elements, and bacteriophages. A previous study reported gene loss in SEZ strains, and gene gain was demonstrated through the acquisition of mobile genetic elements, which shaped the host tropism and pathogenic characteristic of *S*. *equi* [1,54]. The acquisition of prophages plays a vital role in the pathogenicity and diversity of many bacteria [55], and chromosomally integrated prophages, especially, have a much greater influence on the virulence. Presently, more articles on the integration of prophages into the genomes of different bacterial isolates have been reported [56,57,58]. Previous studies have described that several group A *streptococci* prophage-encoded virulence factors were found in group C *streptococci*, like *S. equi* subspecies *equi* and *zooepidemicus* [59,60,61], and were nearly identical to GAS homologs, suggesting phage-mediated horizontal transfer. A previous study reported that the Sz 35246 and Sz H70 strains contain prophages and/or integrative and conjugative elements. But these prophages in SEZ genomes do not carry virulence factors [1]. In the present study, the genomes of ZHZ 211 were aligned and compared to the genomes of Se 4047, XJ 5012, and Sz H70 to examine the genome architecture, chromosomal sequences, and gene inventories, including the mobile elements. Strain ZHZ 211 has one prophage (Ph 01), which carries virulence factors (hyaluronidase). Our finding that Ph 01 was located in the chromosome of ZHZ 211 provided evidence that ZHZ 211 may potentially adapt to diverse environments through the acquisition of this mobile genetic element (Ph 01).

*S. zooepidemicus* has been reported as a hyaluronic acid (HA) producer, and HA capsules not only help the bacteria migrate through the epithelial layers [62] but also protect them from the immune systems of hosts [63]. Hyaluronidase is a secreted enzyme that breaks down HA and chondroitins that are present in the cement substance of host tissues, facilitating invasion by bacteria and their toxins [64]. Therefore, it has been regarded as a spreading factor that plays a vital role in the pathogenic life of *streptococci.*

Previous studies have reported that the SzH70 genome contains a putative hyaluronidase (SZO06680) encoding CDS, and that strain Se4047 contains two hyaluronidases. More interestingly, they found that Se4047 has acquired a different hyaluronidase (SEQ2045), which contains a 4 bp deletion and is located on a prophage. However, in our study, the *S. zooepidemicus* ZHZ 211 and *S. equi* XJ 5012 strains contained two hyaluronidase genes and one hyaluronidase gene, and they were both encoded on prophages. Moreover, according to our result, the hyaluronidase-encoding genes (1905 bp and 1101 bp) of ZHZ 211 were different from those of XJ 5012 (1119 bp) [13]. 

The Type-IV secretion system plays an important role in the virulence and survival of some bacterial species. The Type-IV secretion system comprises 11 structural protein subunits, namely, VirB1–VirB11, the coupling protein VirD4/TraG, and the DNA-processing enzyme VirD2. It has been shown that VirD4-like proteins (TraG) exhibit NTPase activity, facilitating their interaction with both dsDNA and ssDNA [65,66,67], as well as DNA binding without sequence specificity [68,69]. VirD4 may act as an adapter protein and presumably elicits host proinflammatory responses [70]. Previous studies have shown that *virD4* plays a vital role in antiphagocytosis and enhances the release of proinflammatory cytokines, and that the deletion of *virD4* decreased the virulence of *Streptococcus suis* type 2 in a mouse model [71]. Zhang et al. reported that a new T4SS subgroup (Type-IVC secretion system) is located in the 89 K pathogenicity island of *S. suis* type 2 strains [72]. In the present study, two *virD4* genes of the Type-IV secretion system and two transposons (Tns) were identified on one genomic island (GI) of ZHZ 211. In our cases, for the first time, two transposons and two *virD4* genes were reported in a SEZ strain. As previous studies have stated, the acquisition of mobile genetic elements, like prophages or integrative and conjugative elements, enhances the ability of SEZ to adapt to diverse environments and infect a wide range of hosts. Here, we hypothesize that these mobile genetic elements (prophages and transposons) might make an important contribution to the formation of a broad host range of more virulent SEZ strains by introducing virulence genes (*hyl* and *virD4*).

## 5. Conclusions

We conducted a genomic analysis on *S. zooepidemicus* isolates. A mobile genetic element, prophage Ph 01, was determined to be in the *S. zooepidemicus* ZHZ 211 genome that mediates hyaluronidase activity. Interestingly, two transposons and two *virD4* genes were found to be located in one genome island of SEZ strains. Our results showed that the ZHZ 211 isolate is resistant to clarithromycin, enrofloxacin, and sulfonamides and has higher biofilm-forming capabilities. This study provides insights into the genomic features and pathogenicity of the *S. zooepidemicus* ZHZ 211 isolate. The genome will be used as a resource for additional research that may reveal new information on the pathogenic potential and relative health risks posed by *S. zooepidemicus* strains.

## Figures and Tables

**Table 1:** Antibiotics resistance genes and phenotype results of ZHZ 211.

Antibiotics	ZHZ 211	Genes	XJ 5012	Genes
Amoxicilin	S	*PBP2x*, *PBP1a*, *soxS*, *PBP2b*	S	*PBP2x*, *PBP1a*, *soxS*, *PBP2b*
Ampicillin	R	*PBP2x*, *PBP1a*, *soxS*, *PBP2b*	S	*PBP2x*, *PBP1a*, *soxS*, *PBP2b*
Cefuroxime	R	*PBP2x*, *PBP1a*, *soxS*, *PBP2b*	R	*PBP2x*, *PBP1a*, *soxS*, *PBP2b*
Ceftiofur	S	*PBP2x*, *PBP1a*, *soxS*, *PBP2b*	S	*PBP2x*, *PBP1a*, *soxS*, *PBP2b S*
Cefoxitin	S	*PBP2x*, *PBP1a*, *soxS*, *PBP2b*	S	*PBP2x*, *PBP1a*, *soxS*, *PBP2b*
Penicillin	R	*PBP2x*, *PBP1a*, *soxS*, *PBP2b*	S	*PBP2x*, *PBP1a*, *soxS*, *PBP2b*
Gentamicin	S	*vgaE*, *lmrP*, *rpsL*, *cfrA*, *baeS*, *lsaC*, *salA*, *baeS*, *gidB*	R	*vgaE*, *lmrP*, *gidB*, *rpsL*
Streptomycin	S	*vgaE*, *lmrP*, *rpsL*, *cfrA*, *baeS*, *lsaC*, *salA*, *baeS*, *gidB*	S	*vgaElmrP*, *gidB*, *rpsL*
Erythromycin	S	*macBlmrP*, *cfrA*, *mefE*, *efrA*, *efrB*, *mtrA,*	S	*macB* *lmrP, efrA, mtrA, mefE, efrB*
Clarithromycin	R	*macBlmrP*, *cfrA*, *mefE*, *efrA*, *efrB*, *mtrA*	R	*macBlmrP*, *efrA*, *mtrA*, *mefE efrB,*
Doxycycline	S	*macBlmrP*, *cfrA*, *mefE*, *efrA*, *efrB*, *mtrA*	R	*macBlmrP*, *efrA*, *mtrA*, *mefE efrB,*
Oxytetracycline	S	*lmrP*, *tetB(P)*, *adeR*, *soxS*, *rpsJ*	S	*lmrP*, *soxS*, *adeR*, *tetB(P)*, *tetT*, *rpsJ*
Tetracycline	S	*lmrP*, *tetB(P)*, *adeR*, *soxS*, *rpsJ*	R	*lmrP*, *soxS*, *adeR*, *tetB(P)*, *tetT*, *rpsJ*
Levofloxacin	S	*patB*, *arlR*, *arlS*, *gyrA*, *efrA*, *efrB*, *soxS*	S	*efrA*, *arlR*, *arlS*, *soxS*, *efrB*, *gyrA*, *patB*
Norfloxacin	S	*patB*, *arlR*, *arlS*, *gyrA*, *efrA*, *efrB*, *soxS*	S	*efrA*, *arlR*, *arlS*, *soxS*, *efrB*, *gyrA*, *patB*
Enrofloxacin	R	*patB*, *arlR*, *arlS*, *gyrA*, *efrA*, *efrB*, *soxS*	R	*efrA*, *arlR*, *arlS*, *soxS*, *efrB*, *gyrA*, *patB*,
Ciprofloxacin	S	*patB*, *arlR*, *arlS*, *gyrA*, *efrA*, *efrB*, *soxS*	S	*efrA*, *arlR*, *arlS*, *soxS*, *efrB*, *gyrA*, *patB*
Sulfafurazole	R	*/*	S	*/*
Sulfadiazine Sodium	R	*/*	S	*/*
Rifampin	S	*efrA*, *efrB*, *soxS*, *rpoB*	S	*efrA*, *soxS*, *efrB*, *rpoB*
Clindymycin	S	*lmrP*, *cfrA*, *lsaC*, *salA*, *lmrB*	S	*lmrP*, *lmrB*

## Data Availability

The datasets generated for this study can be found in the NCBI GenBank under accession numbers CP133950 (ZHZ 211) and CP134538 (XJ 5012).

## Abbreviations

*hyl*: hyaluronidase; SEZ: *Streptococcus equi* subsp. *Zooepidemicus*; MGEs: mobile genetic elements; WGS: whole-genome sequencing; GI: genome island; COG: Cluster of Orthologous Group; PCOA: principal component analysis; CPS: capsular polysaccharide biosynthesis; THB: Todd Hewitt broth; HGT: horizontal gene transfer; HA: hyaluronic acid; BRIG: Blast Ring Image Generator.
